# Enhancing Sustainability and Antifungal Properties of Biodegradable Composites: Caffeine-Treated Wood as a Filler for Polylactide

**DOI:** 10.3390/ma17030698

**Published:** 2024-02-01

**Authors:** Aleksandra Grząbka-Zasadzińska, Magdalena Woźniak, Agata Kaszubowska-Rzepka, Marlena Baranowska, Anna Sip, Izabela Ratajczak, Sławomir Borysiak

**Affiliations:** 1 Institute of Chemical Technology and Engineering, Poznan University of Technology, Berdychowo 4, 60-965 Poznan, Poland; aleksandra.grzabka-zasadzinska@put.poznan.pl (A.G.-Z.); kaszubowskaagata@gmail.com (A.K.-R.); 2 Department of Chemistry, Poznan University of Life Sciences, Wojska Polskiego 75, 60-625 Poznan, Poland; magdalena.wozniak@up.poznan.pl (M.W.); izabela.ratajczak@up.poznan.pl (I.R.); 3 Department of Silviculture, Poznan University of Life Sciences, Wojska Polskiego 42, 60-625 Poznan, Poland; marlenab@up.poznan.pl; 4 Department of Biotechnology and Food Microbiology, Poznan University of Life Sciences, Wojska Polskiego 48, 60-625 Poznan, Poland; anna.sip@up.poznan.pl

**Keywords:** polylactide, biocomposites, wood, filler modification, caffeine, structure, biological resistance

## Abstract

This study investigates the suitability of using caffeine-treated and untreated black cherry (*Prunus serotina* Ehrh.) wood as a polylactide filler. Composites containing 10%, 20%, and 30% filler were investigated in terms of increasing the nucleating ability of polylactide, as well as enhancing its resistance to microorganisms. Differential scanning calorimetry studies showed that the addition of caffeine-treated wood significantly altered the crystallization behavior of the polymer matrix, increasing its crystallization temperature and degree of crystallinity. Polarized light microscopic observations revealed that only the caffeine-treated wood induced the formation of transcrystalline structures in the polylactide. Incorporation of the modified filler into the matrix was also responsible for changes in the thermal stability and decreased hydrophilicity of the material. Most importantly, the use of black cherry wood treated with caffeine imparted antifungal properties to the polylactide-based composite, effectively reducing growth of *Fusarium oxysporum*, *Fusarium culmorum*, *Alternaria alternata*, and *Trichoderma viride*. For the first time, it was reported that treatment of wood with a caffeine compound of natural origin alters the supermolecular structure, nucleating abilities, and imparts antifungal properties of polylactide/wood composites, providing promising insights into the structure-properties relationship of such composites.

## 1. Introduction

An increasing interest in ecological products requires new, effective, and sustainable materials that reduce global pollution but also have additional user-friendly features. Polylactide (PLA) is a well-known biodegradable polymer that not only gets a lot of attention in the scientific world, but is also becoming more popular among consumers. Although biocompatible and biodegradable, PLA often does not provide the application properties required by users. One way out of this problem is to prepare composite materials. Therefore, many attempts have been made to produce fully biodegradable, sustainable, and satisfactory PLA-based composites in terms of functional properties.

PLA/wood composites are an emerging group of these kinds of materials. For this purpose, numerous species and sources including pine wood [1], hardwood and softwood pulps [2,3], wood flour [4], blends of aspen, oak, pine, basswood [5], and sawdust [6] have been used. Of course, the environmental impact of PLA/wood composites depends on various factors, such as the source of the wood. The use of non-native invasive species may also be an interesting solution to this problem [7]. Given the high prices of PLA, the use of relatively inexpensive by-products of wood processing and wood sourced from responsible forestry (removing invasive plant species) is believed to be more profitable from an economic point of view.

Composites of PLA and wood offer some attractive properties. They were found to be a waste source for the production of high quality 3D polymeric materials [6], reduce production costs, and compensate for the deficiencies of PLA [3,5,8,9]. Despite these obvious advantages, there are also some important issues regarding the use of wood in polymer composites. Achieving a uniform dispersion of wood particles within the PLA matrix may be challenging, and non-homogenous dispersion can lead to weak points, reducing the overall strength and performance of the composite. Moreover, wood tends to absorb moisture, which can lead to swelling, dimensional instability, and susceptibility to microbial attack or decay. Another aspect is ensuring good compatibility and adhesion between the hydrophilic nature of wood fibers and the hydrophobic nature of PLA, crucial to maintaining the mechanical properties of the composites [10,11]. This is also associated with the formation of a proper supermolecular structure of the material. The nucleating abilities and structural aspects influence the mechanical properties of the composite and the enhanced crystalline structure often results in improved mechanical performance, increased heat resistance, and barrier properties [12,13].

The main advantage of PLA composites with wood filler is their biocompatibility and biodegradability under composting conditions. However, this is also their disadvantage: PLA-based materials lack biological protection and thus, are prone to destruction by microorganisms, also during shelf life [14].

There are several strategies that are employed to enhance the biological resistance of PLA/wood composites against microorganisms. Busolo et al. [15] demonstrated the effectiveness of PLA compounds with silver-based engineered clays against *Staphylococcus aureus*. Harnet et al. [16] further improved this protection by using multilayer films made of polyethylene-functionalized chloroethylene on adhesive materials to inhibit the spread of *Escherichia coli*. The scope of antimicrobial protection was expanded by modifying the PLA surface with water-resistant polymer brushes, which significantly inhibited bacterial adhesion [17]. Finally, Ahmed et al. [18] explored the use of plastic PLA films based on essential oils to suppress *S. aureus* and *Campylobacter jejuni*, with cinnamon and clove oil films showing the highest antimicrobial activity. These approaches can be grouped into the following types: (a) chemical treatments that include applying fungicides, biocides, or other chemical treatments that can help inhibit microbial growth and decay in the composite material; (b) natural antimicrobials like essential oils, extracts from plants, or other biobased substances added to the composite materials that can deter microbial attack; (c) surface coatings with antimicrobial properties applied to the surface of composites that can provide an extra layer of protection against microorganisms; (d) nanoparticles with antimicrobial properties; (e) filler modifications through processes like acetylation, heat treatment, or chemical modification [19,20]. The last method mentioned, chemical modification of wood seems to be particularly interesting. Depending on the reagents used, it can be relatively inexpensive and enhance the resistance of the composite to decay and microbial attack.

Caffeine has been found to be an effective and environmentally friendly wood protection agent against decay, fungi, and termites [21,22]. Caffeine has been found to interact with wood components with varying degrees of binding strength depending on the specific component. These interactions are influenced by the composition of wood hemicellulose, with certain sugar monomers and polymers showing higher binding potential [23]. Caffeine treatment at a concentration of 2% has been shown to be effective against wood-destroying fungi and at 1% in some cases [21]. It has also been found to improve the fungistatic properties of thermally modified pinewood [22]. 

Understanding and optimizing all of the above-mentioned aspects of PLA composites is essential for tailoring their properties to meet specific application requirements, ensuring better performance and functionality of the material in various industries such as packaging, biomedical devices, automotive components, and more. In an ideal situation, the filler used for PLA composites should not only enhance the crystallization process, but also provide antimicrobial protection. The use of caffeine-treated wood as a filler for a PLA matrix has not yet been reported and shows great economic and ecological potential. Consequently, the objective of this investigation was to determine whether the addition of caffeine-treated or untreated black cherry wood, a nonnative invasive wood species in Polish ecosystems, has a positive influence on the nucleating behavior of PLA composites and can provide antifungal protection against bacteria and fungi. 

## 2. Materials and Methods

### 2.1. Materials

Polylactide (PLA) 2500HP (Nature Works, Blair, NE, USA) was used as a polymer matrix. The content of D-lactide in this PLA is 0.5% [24]. The wood material was obtained from black cherry (*Prunus serotina* Ehrh.) shrubs collected in 2022, in the Arboretum in Zielonka (52°33′18.2″ N 17°05′49.7″ E). The wood particles had sizes between 0.5 and 1.0 mm. Caffeine in form of powder with purity ≥ 99% (Sigma Aldrich, Darmstadt, Germany) was used for wood treatment. 

### 2.2. Wood Treatment

Black cherry wood was treated with caffeine as previously described in the paper by Tomczak et al. [25]: a 2% aqueous solution of caffeine was prepared and mixed with wood at 20 °C for 2 h. After treatment, the wood was dried until a constant mass was achieved.

### 2.3. Microorganism Preparation

The antimicrobial activity of composite samples was assessed against the microorganisms listed in Table 1. Before the test, the bacterial strains were cultured in nutrient broth (Oxoid, UK) at a temperature of 35 ± 2 °C for 24 h. Next, petri dishes with Mueller-Hilton Agar (Graso Biotech, Owidz, Poland) were inoculated with a suspension of these cultures at a concentration of 10^6^ CFU/mL. In the antifungal test, discs with a diameter of 10 mm were cut from the 7-day-old plate fungal cultures grown in YGC (Yeast Extract Glucose Chloramphenicol) medium and placed in the center of petri dishes with Sabouraud Dextrose Agar (Graso Biotech, Poland).

### 2.4. Preparation of Composites

Before any processing, the materials were first dried in a convection dryer at 65 °C for at least 12 h.

Polylactide composites with wood filler were obtained using a two-step process. First, the corotating twin screw extruder was used. The diameter of the screws was 16 mm and the L/D ratio was 40. The compression ratio, that is, the height of the screw channel in the feeding zone to its height in the dosing zone, was 2:1. The temperatures of the extruder zones were in the range of 190–205 °C. In this process, the filler was introduced into the dosing zone using an external hopper at a speed of 8 rpm. The speed of the extruder screws was 80 rpm. The composites were extruded through an extrusion head with a circular nozzle with a diameter of 3 mm, cooled on a silicone conveyor, and then cut to obtain granules.

The second processing step included injection molding. An ENGEL 80/20 HLS injection molding machine (Schwertberg, Austria) was used. The clamping force of the machine was 200 kN and the diameter of the screw was 22 mm. The temperatures of the injection molding machine zones were set in the range of 190–210 °C. The injection speed was 75 mm/s, the pressing time was 6 s, and the cooling time was 35 s. A single-cavity mold was used as a cold runner with a square socket with dimensions of 100 mm × 100 mm × 2.6 mm (length × width × thickness). An external cooling unit was used to maintain the mold temperature at 35 °C. At least 10 specimens of each composite were molded.

**Figure 1:**
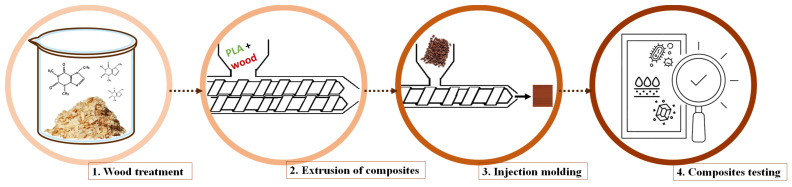
Workflow of the research.

A diagram of the workflow carried out in this research is presented in Figure 1 and the composites prepared in this study are presented in Table 2.

### 2.5. X-ray Diffraction

The supermolecular structure of the materials was analyzed by means of wide angle X-ray scattering—XRD (Rigaku, Tokyo, Japan). CuKα radiation at 40 kV and 30 mA anode excitation was used. The XRD patterns were recorded for 2θ angles from 5 to 40° in steps of 4°/min. The diffraction patterns were used to define the supermolecular structure of the materials, including the polymorphic form of the fillers used. The characteristic peaks were assigned to respective crystal planes. 

### 2.6. Differential Scanning Calorimetry

The thermal characteristics of the produced composites were assessed through differential scanning calorimetry (DSC) using a Netzsch DSC 200 instrument (Netzsch, Bavaria, Germany), operating in a nitrogen atmosphere. To conduct non-isothermal crystallization studies, the samples underwent an initial heating phase from 40 to 200 °C at a rate of 20 °C/min, followed by a 3-min dwell at this temperature to eliminate any prior thermal or mechanical influences. Subsequently, the samples were cooled to 40 °C at a rate of 5 °C/min and held at this temperature for 1 min. This entire procedure was repeated. The enthalpy of the crystallization (H) values determined were employed to calculate the degree of crystallization (crystal conversion), denoted as α, using Equation (1):

(1) $$\alpha =\frac{\int_{0}^{t}(\frac{dH}{dt})\times dt}{\int_{0}^{\alpha }(\frac{dH}{dt})\times dt}$$

Based on curves of α = f(t), the half-time of crystallization (t0.5) was determined when the crystal conversion reached 50%. The degree of crystallinity (X_c_) of the materials was calculated according to Equation (2).

(2) $${\;X}_{c}=\left(\frac{∆Hm-∆Hcc}{∆Hm{}^{\circ}\times (1-\frac{\%wt\;filler}{100})}\right)\times 100$$

where: ΔHm is the melting enthalpy, ΔHcc is the enthalpy of the phase produced during cold crystallization, ΔHm° is the melting enthalpy of a 100% crystalline polylactide (93.0 J/g [26]) and %wt. filler is the percentage of filler weight.

In addition, the melting (Tm), crystallization (Tc), and cold crystallization (Tcc) temperatures were defined.

### 2.7. Fourier Transformation Infrared Spectroscopy

Untreated and caffeine-treated wood were mixed with KBr (Merck KGaA, Darmstadt, Germany) at a 1:200 weight ratio and analyzed in the form of pellets using a Nicolet iS5 Fourier transform spectrophotometer (Thermo Fisher Scientific, Waltham, MA, USA).

### 2.8. Thermogravimetric Analysis

Thermogravimetric curves for the composites (samples of approximately 10 mg) were obtained using a NETZSCH TG 209F3 apparatus (Netzsch, Germany). Measurements were carried out at a heating rate of 10 °C/min over the temperature range of 50 to 500 °C under nitrogen flow (20 mL/min).

### 2.9. Polarized Light Microscopy

The isothermal crystallization of the PLA in the presence of untreated and treated wood was conducted using a Linkam TP93 hot stage optical microscope (Linkam, Redhill, UK) and a Nikon Eclipse polarizing optical microscope, model LV100POL (Nikon, Melville, NY, USA) equipped with a Panasonic CCD GP-KR222 camera (Panasonic, Newark, NJ, USA).

The PLA granules were cut into small pieces (ca. 30 μg). One piece was placed on a microscopic glass and then a few particles of filler were strategically placed on its surface. The samples underwent initial heating to 200 °C at a rate of 20 °C/min and were maintained at this temperature for 3 min to erase any prior thermal and/or mechanical effects. After melting, the sample was pressed with another microscopic glass. The samples were then cooled at 10 °C/min to 136 °C, at which point isothermal crystallization of the PLA was possible. Photographs were taken every 5 s and analyses were performed using the ToupView program (ToupTek, Hangzhou, China). On that basis, the size and growth rate of the PLA spherulites was determined. 

### 2.10. Contact Angle Measurements

The measurements of the contact angle were performed using a Dataphysics OCA 200 instrument (DataPhysics Instruments, Filderstadt, Germany). All measurements were carried out at 21 ± 0.5 °C. A drop of 0.2 mL of water was dispensed from the capillary in a controlled manner and placed onto the solid surface of the sample.

### 2.11. Antimicrobial Properties

PLA-based samples (sterilized by autoclaving at 121 °C for 15 min) of approximately 1 × 1 cm were put on petri dishes with Mueller-Hilton Agar (Graso Biotech, Poland) and Sabouraud Dextrose Agar (Graso Bioetch, Poland) inoculated with bacterial or fungal strains. The samples tested with bacteria were incubated at 35 ± 2 °C for 24 h, while the samples tested with fungi were kept at a temperature of 30 ± 2 °C for 28 days. After incubation, the antimicrobial activity of the tested materials was assessed. For this purpose, measurements of the inhibition zones formed around the polymeric samples were made. Measurements were performed using a Computer Scanning System (MultiScaneBase v14.02). The results were expressed in millimeters. 

For the antifungal activity testing, mold growth was assessed on the 7th, 14th, 21st, and 28th day of incubation. It was also observed whether the mold grew on the tested samples. The degree of colonization of the materials tested by fungi was determined according to the four-level scale presented in the paper by Tomczak et al. [25]. 

In the second set of antimicrobial experiments, the test samples were placed on the surface of nutrient agar (Oxoid, UK) and then covered with Mueller-Hilton Agar medium (Graso Biotech, Poland) inoculated with 10^6^ CFU/mL of the tested bacterial strains. The plates were incubated under the same conditions as in the first series of experiments. The antibacterial activity of the tested materials was also evaluated in a similar way.

## 3. Results and Discussion

### 3.1. Structure of Lignocellulosic Materials

In the first step, black cherry wood fillers (both untreated and caffeine-treated) used for the formation of composites were tested in terms of supermolecular structure, using the XRD method. 

**Figure 2:**
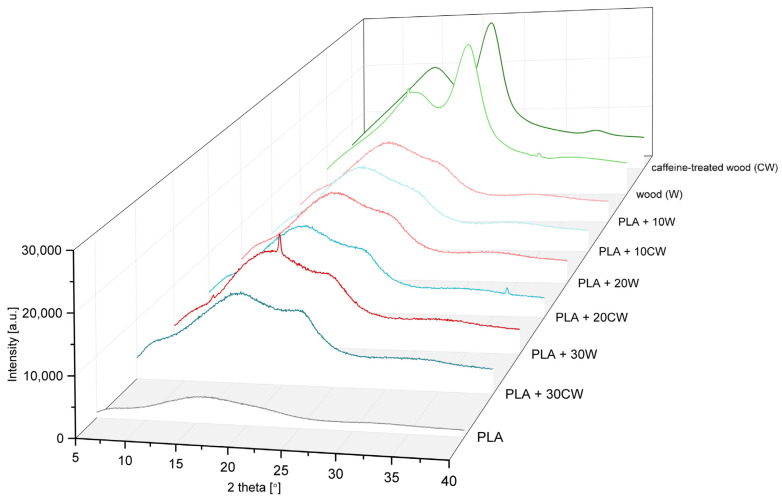
Diffractograms of fillers, PLA matrix and composites.

As shown in Figure 2, both fillers exhibited a typical structure for cellulosic materials: the wide peaks were present at 2θ 15.7° and 22.5°. However, in untreated wood, some smaller peaks, around 15° and 30°, were observed that may be a result of the presence of substances of low molecular weight naturally occurring in wood [27]. These peaks were not registered on the diffractogram of the caffeine-treated wood, meaning that they had been removed during treatment. The crystallinity degree of both types of filler was comparable, around 25%.

Then, PLA and its composites were tested. The obtained diffraction patterns are also presented in Figure 2.

The diffractogram of PLA shows a wide amorphous halo with a maximum intensity around 2θ = 16°, but no crystalline peaks were present. In all composite samples, the amorphous halo was present, but only in the sample containing 30% of untreated wood was a small crystalline peak at 2θ = 16.3° present. It corresponds to the planes (110) and (200) of the ortho-rhombic α-crystalline phase of PLA [28]. Wood is known to be a nucleating agent [29,30], but in this case, it is rather a result of composite processing—at a higher filler loading, shear forces occurring during injection molding are higher than in case of lower filler composites, enhancing the crystallization process [31,32]. A wide maximum around 2θ = 22.5°, more pronounced for composites with a high content of wood (un- and treated), resulted from the presence of cellulose, a component of lignocellulosic mass [30].

**Figure 3:**
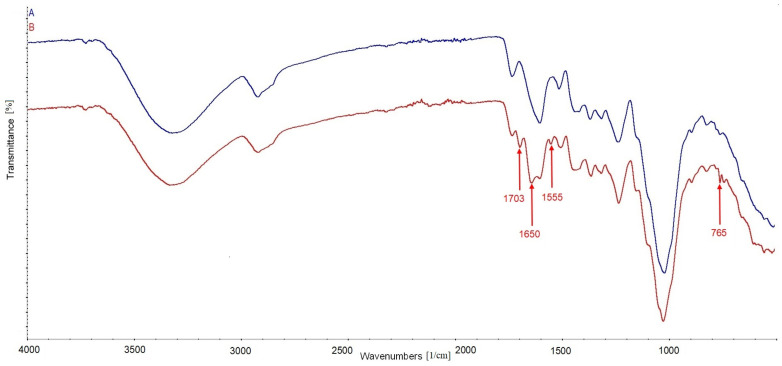
Spectra of untreated cherry wood (A) and cherry wood treated with caffeine (B).

The treatment of cherry wood with caffeine caused changes in its chemical structure, which was confirmed by FTIR analysis, presented in form of spectrum in Figure 3. 

The most important changes in the spectrum of treated wood compared to the spectrum of untreated wood confirm the presence of bands characteristic for caffeine molecules. The peaks at 1703 and 1650 cm^−1^ can be attributed to the vibrations of C=O and/or the N-H bonds of the acetamide group of amide I [25,33]. In turn, the peaks at 1555 and 765 cm^−1^ can be ascribed to the vibration of the N–H and/or C–N groups of amide II [34,35]. 

### 3.2. Nucleation Ability of Wood Fillers and Crystallization Process in Composites

**Figure 4:**
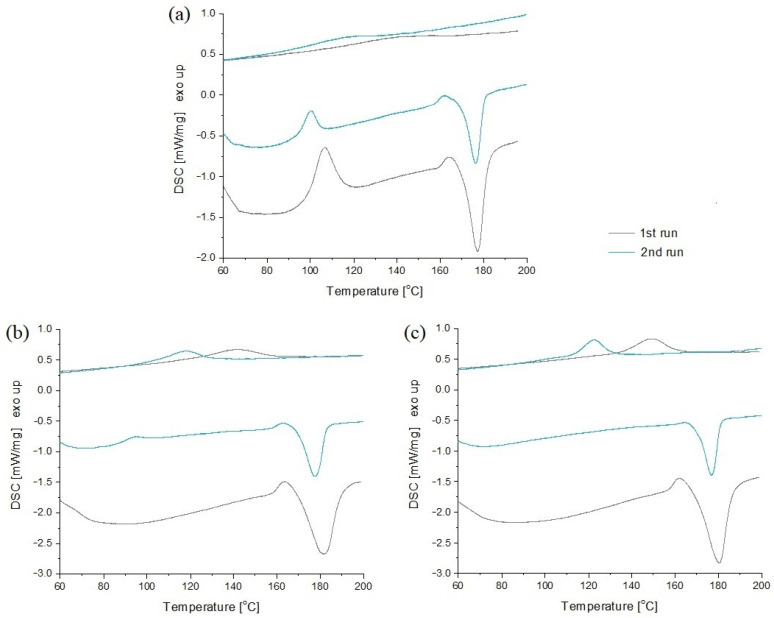
Exemplary thermograms of: (**a**) PLA, (**b**) PLA + 20 W, and (**c**) PLA + 20 CW.

A differential scanning calorimetry study was carried out to determine the phase transitions that take place in polylactide in the presence of a filler, un- and treated with caffeine. As a result of the experiment, thermograms showing heating and cooling curves were obtained. Exemplary thermograms of PLA and composites are shown in Figure 4, while all data collected from the second runs are summarized in Table 3.

For polylactide, cold crystallization and melting peaks were observed, but no crystallization occurred during cooling. This is the typical behavior of this polymer, which was also described in other papers. The temperatures at which the phase transitions occurred were also in line with those of the literature [36,37].

The presence of cold crystallization is a characteristic feature of PLA. The fact that PLA does not fully crystallize during cooling makes it possible that during subsequent heating, below the melting point, disordered polymer chains can rearrange into ordered crystalline structures. The degree and rate of cold crystallization in PLA varies according to the cooling rate, processing conditions, molecular weight, and the presence of additives and nucleating agents [38]. 

In the case of composites, the cold crystallization peak was present in all samples containing untreated wood and in the PLA with 10% CW. It can also be seen that for PLA, the Tcc was greater than 100 °C, while for composites it was in the range of 95.0–98.4 °C. This suggests that the energy required for the sample to recrystallize is lower if it contains filler, thus it enhances the crystallization process.

The addition of untreated wood was not found to have any important influence on the melting temperature, ranging from 176.5 °C to 178.3 °C. It was proven that the stress applied to the polymer melt during processing induces crystallization, which in turn influences the melting temperature. Also, the crystal perfection influences this parameter: the better the formation of crystal structures, the higher the melting temperature [39,40]. Nonetheless, no changes suggesting differences in crystal ideality were observed in the present study. 

The most noticeable changes were observed for the crystallization temperature. The unfilled PLA did not crystallize during cooling at all. This is a well-known fact that affects its application potential. It also shows that the cooling rate of PLA used in this study (5 °C/min) was still too fast to obtain a semicrystalline structure. However, the addition of fillers was favorable, inducing the crystallization process, which took place at 95.9 °C for PLA + 10 W and at around 102.5 °C for PLA with 20 W and 30 W. If caffeine-treated wood was used, the Tc parameter varied from 99.9 °C up to 106.9 °C, for 10 CW and 30 CW, respectively.

When differences in the crystallization behavior of samples are taken into account, it is no surprise that the crystallinity degree parameter was also highly affected by the amount and type of filler used. The degrees of crystallinity calculated indicate that the addition of each type of filler caused an increase of the parameter from 28% for PLA to 45–64% for composites. In composites with treated wood, the following relationship was observed: the higher the wood content, the greater the degree of crystallinity. This is also consistent with the values of Tc. In contrast, in composites with untreated wood, Xc did not depend on the filler content, but it was comparable, in the range of 56–64%. 

The relationship between the crystallinity degree of a polymer composite and the filler content is often complex and may not be strictly proportional. It is a delicate balance between an increase in the number of nucleating points and a decrease in the polymer mobility. Several factors contribute to this nonlinear behavior, and the effect of filler content on crystallinity can vary depending on the specific characteristics of the polymer, the filler, and the processing conditions.

The uneven morphology of caffeine-treated wood offers more nucleating sites than the smooth surface of the wood particles. The number of nucleating sites at which the formation of spherulites begins is higher when more filler is present in the sample. Therefore, the crystallinity degree of the materials increases, which, from a technological point of view, is an important aspect. The ability to achieve a higher degree of crystallinity during a shorter time, without the need to anneal, enables easier production of elements using injection molding, extrusion, or blow molding processes [41,42]. 

**Figure 5:**
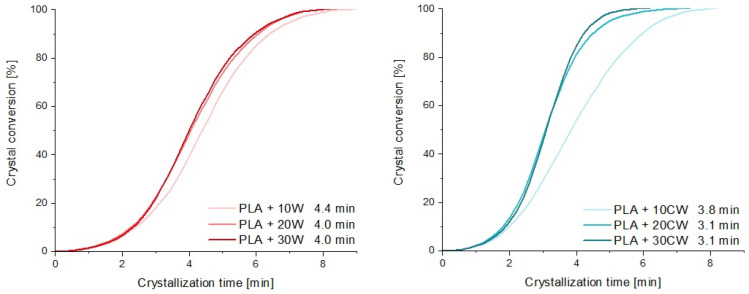
Crystal conversion curves.

On the basis of the thermograms, crystallization half-times were calculated, according to Equation (1). The curves used for calculations along with crystallization half-times are presented in Figure 5.

For composites with untreated fillers, half times were in the range of 4.0–4.4 min, while for modified composites, they were 3.1–3.8 min. It can be concluded that modification of the filler strongly affects the crystallization of PLA, causing a shortening of the conversion time and thus, indicating an acceleration of the crystallization process. There was also a tendency for composites with the lowest amount of filler to have the highest conversion time. 

The results of thermal analysis suggest that the modified filler offers more nucleating sites at which the crystallization process may begin. This is supported by the findings of Tomczak et al. [25], who stated that the surface of caffeine-treated wood is less smooth than that of pristine wood and has some grooves that may facilitate the formation of crystalline structures. This is confirmed by shorter crystallization half-times and higher crystallization temperature values. All of the fillers used were heterogeneous nucleants for the PLA matrix. When high loads of caffeine-treated wood were used (20% and 30%), crystallization during cooling was sufficient and no cold crystallization occurred. Furthermore, the crystallization temperature of the composites differed, reaching the highest values for PLA + 20 CW and PLA + 30 CW, once again proving the nucleating effect. 

**Figure 6:**
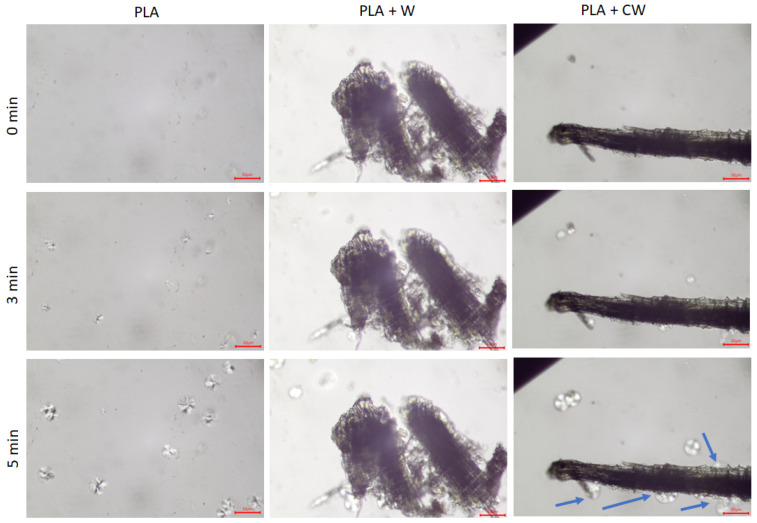
PLM photographs of unfilled PLA and PLA with wood and caffeine-treated wood. The scale bar is 50 μm.

A study was carried out using a polarizing microscope to check the effect of the filler on the crystallization of polymer matrix. Photos taken during the experiment under isothermal conditions are shown in Figure 6.

For the unfilled PLA matrix and PLA with untreated wood, the crystallization process took place in bulk. However, in the composite samples with treated filler (PLA + CW), the formation of spherulites was observed not only in bulk, but also on the surface of the caffeine-treated wood. Interestingly, the use of that filler resulted in the formation of transcrystalline structures, TCL (shown with blue arrows). In addition, for the PLA + CW samples, a larger number of spherulites were formed. This suggests that this type of filler was a better nucleant for the polylactide matrix.

Observations made by means of microscopy support the findings of the DSC analysis (especially the conversion rate and crystallization temperature) that showed that caffeine-treated wood is an active nucleant for PLA. As a result, a well pronounced formation of the transcrystalline layer was observed. This may be the result of the removal of low-mass molecules present in untreated wood and, therefore, the incorporation of some roughness on the surface of the filler. The following analysis of the literature shows that surface topography may affect the nucleation of polymers in two main ways. First, the thermal stress that develops at the filler/polymer interface might induce the local orientation of the polymer chain segments, which enables easier nucleation. Second, the nucleation rate of the composite was found to be determined by changes in the free energy barrier and the diffusion activation energy under different interfacial interactions [43]. Crystallization on a smooth surface requires a higher energy, and when grooves are present, the free energy barriers necessary to initiate the nucleation process are lower [44,45].

### 3.3. Thermogravimetric Analysis

**Figure 7:**
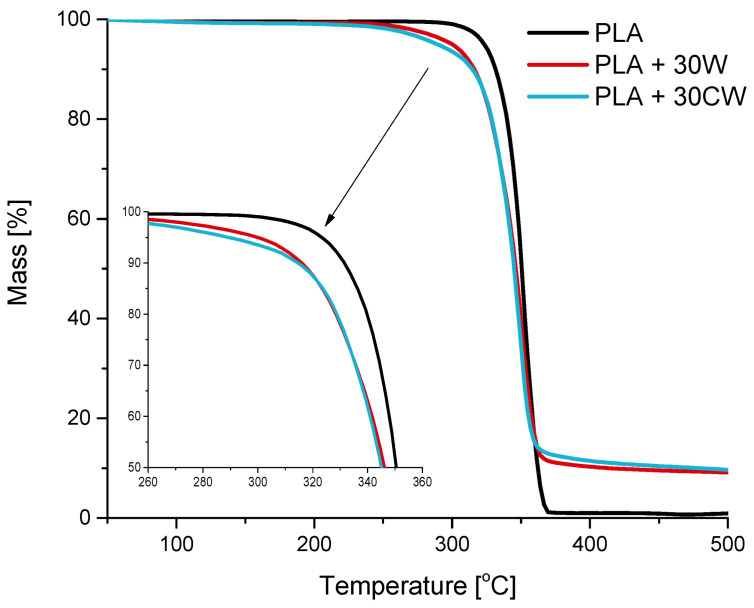
Thermogravimetric curves for PLA and composites with 30% filler.

A thermogravimetric analysis of PLA and composite samples was performed to define the thermal stability of the samples. The recorded thermograms are shown in Figure 7, while Table 4 summarizes the temperatures at which 10%, 50%, and 90% of the mass of the samples were lost, along with their char content.

All of the TG curves had similar shapes. The decomposition of the samples occurred in one step, with a characteristic inflection of the curve at around 300 °C and at approximately 360 °C. These are values typically observed for PLA. During its thermal decomposition, lactide is released first and then the higher cyclic oligomers [46]. 

Taking into consideration the temperatures at which 10% and 50% mass loss occurred, it can be seen that the values observed for the composites were slightly lower than those observed for the unfilled samples, especially at the first threshold. Other studies reported similar observations about PLA with wood fibers [47,48] and wood flour [49]. This behavior is ascribed to the presence of wood and its main constituent, cellulose, which begins to degrade at lower temperatures [50].

At a mass loss of 90%, there was a large difference between the PLA, PLA + 30 W, and PLA + 30 CW samples. The temperature observed for PLA was 48 °C and 109 °C lower than for the composite with wood and treated wood, respectively. This interesting observation can be discussed in terms of wood particles being a physical obstacle to effective heat transfer. The untreated wood present in the composite simply hinders heat transfer. In the case of the modified filler, the caffeine treatment removed some of the low-mass particles present in the wood and thus improved the interaction between the filler and the matrix, making it more difficult for heat to be transferred through the material. Furthermore, the amounts of char present in the residual sample were ten times higher for composites than for PLA. This is in line with other data from the literature [51]. It results from the thermal decomposition of the lignin (one of main constituents of wood) that was found responsible for the formation of char during thermal decomposition of wood, at a temperature over 400 °C [52]. Data analysis also shows that in composites with modified filler less wood was decomposed, proving that caffeine treatment can ‘protect’ wood from heat. 

Overall, what is most important, under typical processing conditions, that is, up to 250 °C, was that the mass loss for both composites did not exceed 2%.

### 3.4. Contact Angle Measurements

**Figure 8:**
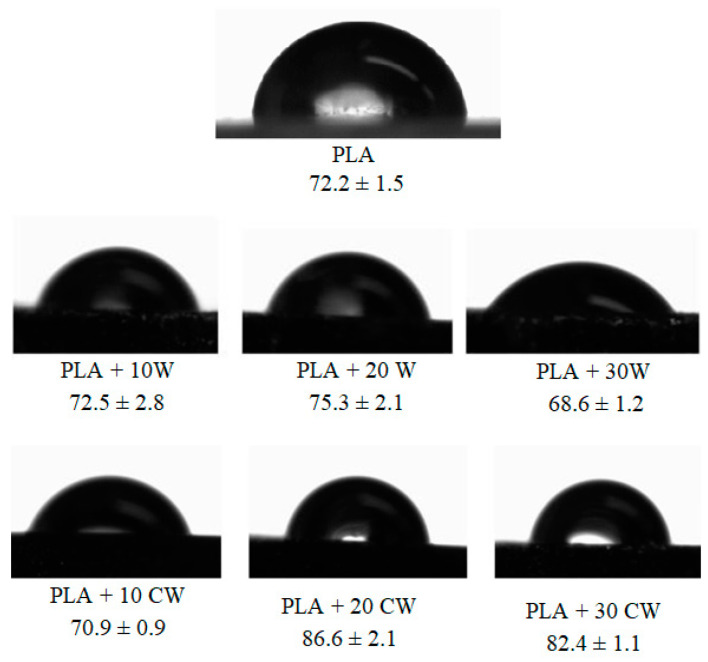
Water drops deposited on samples and calculated, mean values of contact angle.

Contact angle measurements were taken to determine the wettability of the composites obtained. Based on the photos obtained (Figure 8), contact angles were determined.

The contact angle measured for the PLA, 72.2°, is similar to that presented in other articles [13]. It can be seen that the unfilled PLA, as well as all composites, are within the range that classifies materials as hydrophilic. When 10% and 20% of untreated wood is added to the PLA, the composites produced have comparable values of contact angle polymer matrix (68.6–75.3° vs. 72.2° for PLA). A higher content of untreated wood causes the material to be more hydrophilic. This is rather expected because the wood itself is a hydrophilic material. The composite with the smallest amount of caffeine-treated filler had a contact angle similar to that of unfilled polymer wood. On the other hand, increasing the content of modified filler in the composite increased the contact angle. These materials were characterized with lower hydrophilicity than the PLA (contact angle in the range of 82.4–86.6°).

Yet optimistically, these results still remain within the range typical of a hydrophilic material. However, they show that the incorporation of properly modified wood into PLA does not have to result in increasing the hydrophilicity of the material. This may arise from interactions between the constituents of wood and the modifier, caffeine. Treatment of the filler could have resulted in the formation of more hydrogen bonds between cellulose and caffeine molecules [23]. Additionally, research conducted by Tavagnacco et al. [53] confirmed that hemicellulose has a significant affinity for caffeine, which also creates additional bonds.

### 3.5. Antimicrobial Properties

**Figure 9:**
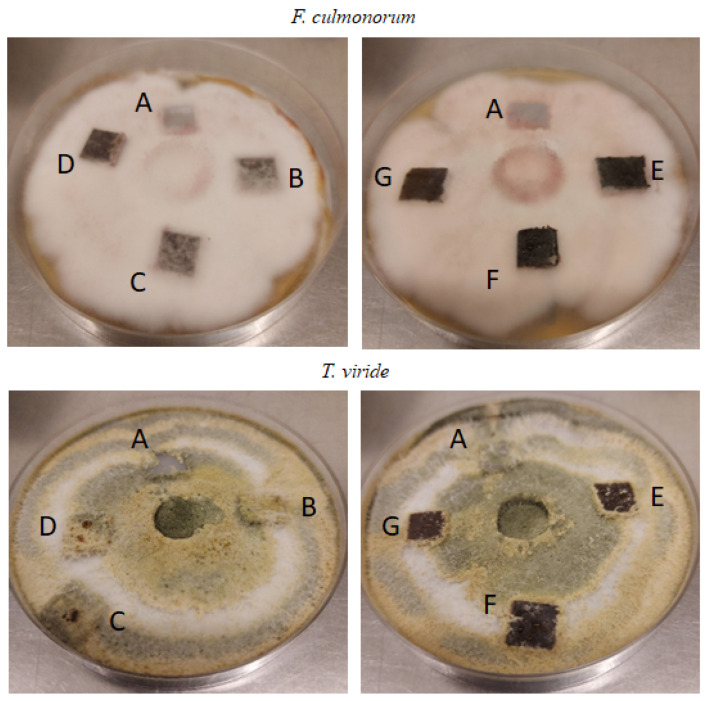
Samples tested against *F. culmonorum* and *T. viride* after 21 days. Samples in figures are named as follows: A: PLA, B: PLA + 10 W, C: PLA + 20 W, D: PLA + 30 W, E: PLA + 10 CW, F: PLA + 20 CW, G: PLA + 30 CW. (*F. culmorum*: over 60% of the surfaces of samples A, B, and C are covered with fungal mycelium; traces of fungal mycelium are visible on the surface of samples D, E, F, and G; *T. viride*: over 60% of the surfaces of samples A, B, C, and D are covered with fungal mycelium; traces of fungal mycelium are visible on the surface of samples E, F, and G).

Antimicrobial tests were performed to assess whether the addition of caffeine-treated wood to the PLA matrix altered the susceptibility of the material to microorganisms. Data regarding antifungal properties are summarized in Table 5 and exemplary photos of samples are shown in Figure 9.

Regardless of the bacterial strain used (*L. innocua* or *L. monocytogenes*), none of the composite samples showed antibacterial activity.

The results presented in Table 5 indicate that the PLA and composites with untreated wood showed no activity against the tested fungal strains. More than 66% of the surface of the PLA and composites with 10% and 20% wood was colonized by *F. culmorum*. Each sample containing caffeine-treated wood and PLA with 30 CW filler showed no mold growth on their surface. A similar behavior was observed for *A. alternata*: all composites with caffeine-treated filler remained unaffected by fungi, while the unfilled PLA and materials with 10% and 20% untreated wood were significantly infected. The composite with 30% wood was also covered with mold, but less so (33% of the sample). All other fungi were responsible for mold growth, but only in the PLA sample and in the composites with untreated wood. The presence of filler and crystallinity degree were reported to affect the biodegradation process of PLA and its susceptibility to microorganisms [54,55,56], but in this study, it was caffeine treatment that affected antifungal properties. 

Caffeine has previously been shown to have an antifungal effect not only in wood but also in polymer composites [21,22,25,57]. However, these previous studies have been performed for wood or wood-polypropylene composites. This is the first study on caffeine-treated wood used as a filler for a PLA matrix, which exhibits antifungal properties. This antimicrobial behavior is attributed to interactions between the cell walls of fungi and caffeine, since caffeine inhibits the synthesis of chitinases, enzymes responsible for the synthesis of cell walls of fungi [58,59]. The interesting thing is that the composite with untreated black cherry wood (PLA + 30 W) also showed antifungal behavior against *F. oxysporum* and *F. culmorum* (Table 5). This may be due to the fact that black cherry is a rich source of phenolic compounds [60] that were found to have antimicrobial properties [61,62].

This is a very important finding, as the fungi used in this research produce toxins (*Fusarium* spp.), cause the spoilage of various raw materials and food products (*A. alternata*), or are used in the biological protection of plants against pathogenic organisms (*T. viride*). As a consequence, these results provide new information that wood-filled composites, based on the bioderived polymer matrix, PLA, can be successfully and relatively easily modified in order to obtain materials that can withstand the negative influence of microorganisms.

## 4. Conclusions

This work deals with the preparation and in-depth characterization of polylactide biocomposites filled with black cherry wood treated with caffeine. This is the first study to show an important relationship between the structure of a polylactide filled with a wood product treated with a natural compound (caffeine) and the performance, including the antifungal properties.

Based on the findings presented in this study, it can be concluded that black cherry wood treated with caffeine is an effective heterogeneous nucleant for polylactide. The degree of crystallinity of composites with caffeine-treated wood was definitely higher than that of PLA, and increased with the wood filler content. The most noticeable changes in the crystallization temperature towards higher values and a reduction in the half-time of crystallization were observed for the modified filler. Unlike untreated wood, the use of a caffeine-treated wood filler resulted in the formation of transcrystalline structures. Interestingly, the introduction of this filler into the polylactide matrix also decreased its hydrophilicity. The experiment demonstrated that the incorporation of black cherry wood treated with caffeine into the polylactide matrix results in a material exhibiting antifungal properties against *F. oxysporum*, *F. culmorum*, *A. alternata*, and *T. viride*.

These findings underscore the potential of black cherry wood, especially when treated with caffeine, as a versatile and multifunctional filler to improve the crystallinity, structure, and antifungal properties of polylactide-based materials. This research contributes valuable information on the development of sustainable and functional biocomposites for diverse applications in the fields of materials science and antimicrobial engineering.

## Figures and Tables

**Table 1:** Microorganisms used as indicators to assess the antimicrobial activity of the tested materials.

Microorganisms	Category	Origin
Gram-positive bacteria		
*Listeria innocua*	non-pathogenic bacteria with strong adhesive properties found in many production plants;	ATCC 33090
*Listeria monocytogenes*	pathogenic bacteria; parasite of animals and humans;	ATCC 19111
Fungi		
*Fusarium oxysporum*	toxin-producing fungi	environmental isolate
*Fusarium culmorum*	toxin-producing fungi	environmental isolate
*Alternaria alternata*	fungi that cause spoilage of various raw materials and food products.	environmental isolate
*Trichoderma viride*	fungi used in biological protection of plants against pathogenic organisms	environmental isolate

**Table 2:** Prepared PLA-based samples.

Polymer Matrix	Filler Type	Filler Content	Sample Name
PLA	-	0%	PLA
	untreated wood	10%	PLA + 10 W
		20%	PLA + 20 W
		30%	PLA + 30 W
	caffeine-treated wood	10%	PLA + 10 CW
		20%	PLA + 20 CW
		30%	PLA + 30 CW

**Table 3:** Summarized data from second runs of DSC.

Sample	Tcc [°C]	Tm [°C]	Tc [°C]	ΔHm [J/g]	Xc [%]
PLA	100.3	176.5	nd	37.39	28
PLA + 10 W	97.8	178.3	95.9	58.72	60
PLA + 20 W	95.0	177.7	102.5	46.23	56
PLA + 30 W	96.5	177.7	102.6	46.65	64
PLA + 10 CW	98.4	177.6	99.9	53.01	45
PLA + 20 CW	nd	176.9	104.9	38.29	52
PLA + 30 CW	nd	176.3	106.9	37.35	58

nd—not detected.

**Table 4:** Temperature at which specific mass loss occurred and char content.

Sample	Temperature [°C]			Char Content at 500 °C [%]
	at 10% Mass Loss	at 50% Mass Loss	at 90% Mass Loss	
PLA	332	350	362	0.9
PLA + 30 W	316	345	410	9.0
PLA + 30 CW	315	347	471	9.8

**Table 5:** Resistance of PLA and its composites to fungi, tested after 21 days.

Fungi	PLA	PLA + 10 W	PLA + 20 W	PLA + 30 W	PLA + 10 CW	PLA + 20 CW	PLA + 30 CW
*F. oxysporum*	2	2	2	0	0	0	0
*F. culmorum*	2	2	2	0	0	0	0
*A. alternata*	2	2	2	1	0	0	0
*T. viride*	2	2	2	2	0	0	0

2: over 66% of the sample covered with fungal mycelium; 1: less than 33% sample covered with mycelium; 0: no signs of fungal mycelium on the sample.

## Data Availability

Data are contained within the article.
